# Effects of vitamin B_12_ supplementation on the quality of Ovine spermatozoa

**Published:** 2013-12-15

**Authors:** M.A. Hamedani, A.M. Tahmasbi, Y.J. Ahangari

**Affiliations:** 1 *Department of Animal Science, Faculty of Agriculture, Ferdowsi University of Mashhad, Mashhad, Iran*; 2 *Department of Animal Science, Faculty of Agriculture, University of Gorgan, Gorgan, Iran*

**Keywords:** *Dallagh ram*, Freezing, Semen characteristics, Vitamin B_12_

## Abstract

The present study was undertaken to investigate the effect of various levels of vitamin B_12_ in Tris extender on semen quality of *Dallagh rams* following the cooling and freeze/thawing process. Semen was collected from six healthy and mature rams with an average body weight of 60.0 ± 5.0 Kg using an electro ejaculator. High quality samples were mixed and diluted in Tris extender supplemented with different concentrations of vitamin B_12_ (0, 1, 2 and 3 mg/ml). The semen aliquots were cooled and preserved at 5°C and their qualities were evaluated during pre-freezing and then the cooled semen samples were packaged into 0.25 ml straws. Straws were frozen in the vapor of liquid nitrogen, and were then stored at -196ºC. Straws were thawed seven days later and the characteristics of spermatozoa were examined. Results of this study showed that the effect of vitamin B_12_ on characteristics such as viability, motility, progressive motility and normality of spermatozoa were significant in pre and post freezing conditions (*P*<0.05). In conclusion, for long term storage of semen of *Dallagh rams*, we recommend using 2 mg/ml of vitamin B_12_ in semen extender.

## Introduction

The freezing and thawing procedure has a significant impact on the survival rate of sperm its after cryopreservation. Cold shock irreversibly decreases both the metabolic activity and sperm motility (Robertson *et al.*, 1990; White, 1993).

Differences among species in the sensitivity of their sperm to cooling are largely attributable to variations in the sperm plasma membrane composition (Drobnis *et al.*, 1993). Unsaturated fatty acids, that predominate in ram sperm membranes, are susceptible to peroxidation (Halliwell and Gutterridge, 1984), thus leading damage of the sperm membrane, inhibition of respiration, and leakage of intracellular enzymes (White, 1993).

Studies to preserve unfrozen semen revealed that controlling oxidation by use of exogenous antioxidants in the extender can, greatly maintain sperm quality (Foote, 1967). Wallock *et al*. (2001) reported that Folic acid (vitamin B_9_) might be vital to proper development of human sperm because it is needed for the production of DNA. Recent analyses of DNA damage in human sperm following exposure to different antioxidant agents have provided some support for this concept (Tremellen, 2008).

Vitamin B_12_ is one of the water-soluble vitamins functioning as a coenzyme in methionine synthesis and metabolism of branched amino acids (Juanchi *et al.*, 2000). Because of its stability, cyanocobalamin is the form typically used in vitamin supplements.

Watanabe *et al*. (2003) reported that Vitamin B_12_ deficiency increased the number of abnormal sperm and decreased the motility and velocity of sperm in male rats. Vitamin B_12_ acts with folic acid and vitamin B_6_ to control homocysteine levels (Juanchi *et al.*, 2000).

In rams, it was suggested that vitamins B_1_, B_6_, and B_12_ play a key role in thermoregulation of scrotal skin and rectal temperature and maintain libido, semen quality, and fertility during heat stress (El-Darawany, 1999). Previous studies have shown that supplementation of antioxidants like cyanocobalamin (vitamin B_12_) to the freezing extender improves the quality of thawed spermatozoa and subsequent fertilization ability in cattle and rams (Ha and zhao, 2003; Dalvit *et al.*, 2005).

However, an excessive addition of vitamin B_12_ into the semen extender can neutralize the oxidative stress via means of excessive ROS formation; it can also arrest the normal sperm functions associated with ROS. Therefore, it is important to select the appropriate antioxidant concentration to maintain the natural balance that exists between ROS generation and scavenging activities. Additionally, there is a lack of detailed information on the effects of supplemental vitamin B_12_ in the extender on frozen semen quality in rams. Therefore, the aim of this study was to evaluate effects of vitamin B_12_ supplementation in various concentrations in ram semen extender on the semen quality during pre and post freezing.

## Materials and Methods

### Animals and Semen Collection

These experiments were performed around Golestan and Mashhad province in Iran. In this study Six *Dallagh rams* with an average age of 3.5 ± 0.5 years and with an average body weight of 60.0 ± 5.0 kg were selected and were housed individually in pens on semi-slatted floors. Animals were fed with a diet according to the recommendations of the National Research Council (NRC) based on 80:20 ratio of forage (alfalfa) to concentrate ad libitum and had free access to water. From each ram, eight ejaculates were collected using electro ejaculator as described by Evans and Maxwell (1987).

### Semen Processing

Semen samples were immediately transferred into graduated test tubes after collection, placed in a thermo flask at 37°C, and transported to the laboratory for evaluation within 1h interval. The raw or fresh undiluted semen was then microscopically evaluated for mass activity, percent motile sperm, progressive motility, viability and abnormality spermatozoa. Semen samples that showed more than 80% motility and viability were selected for this experiment (Table 1).

**Table 1:** Volume, motility wave, motility, progressive motility, viability, abnormality and normal spermatozoa in *Dallagh* *rams*, under fresh condition.

Fresh semen						
Volume (ml)	wave motion	Motility (%)	Progressive Motility (%)	Viability (%)	Concentration (ml)	Normal Spermatozoa (%)
1.2	4.5	85	80	88	3.5. 10^9^	96.3

The semen samples were pooled to eliminate individual differences and divided into four equal aliquots and kept at 37ºC in water bath. After primary observation, semen samples were diluted at a 1:4 ratio (semen: diluent) and concentrations 3.5 × 10^9^ spermatozoa/ml at 37ºC with Tris extender. The dilution contained Tris (hydroxyl methyl) amino methane (3.876g), glucose (0.523 g), citric acid (2.123 g), egg yolk (15%), glycerol (5%), penicillin (100000 IU) and streptomycin (100 mg) (Evans and Maxwell, 1987).

Semen was split into four parts and different amounts of vitamin B_12_ (0 (control), 1, 2 and 3 mg/ml) were added to each group. Diluted semen was cooled gradually to +5ºC within 2 hours. After 2 h, a part of the samples was investigated. The remaining part of the samples was packaged into 0.25 ml straws. At first, straws were frozen at heights of four and six cm above the level of the liquid nitrogen, the frozen straws were then transferred to liquid nitrogen.

After seven days, straws were thawed in a water bath at 37ºC (Maxwell *et al.*, 1995) and then characteristics of motility, progressive motility, viability and normality/abnormality were examined.

### Semen evaluation

### Volume

The volume of the ejaculate was measured by reading the graduated tube (Biswas *et al.*, 2002).

### Mass motility and Motility

Mass motility and motility were evaluated as per the procedures described by Biswas *et al*. (2000) and Bearden and Fuquay (1992), respectively.

### Viability

To evaluate the number of live/dead spermatozoa, eosin nigrosin preparations were prepared according to the method described by Bearden and Fuquay (1992). A total of 100 sperm cells were counted on each slide at 400X magnification.

### Progressive motility

A drop of semen diluted in 1:4 ratio with Tris was placed on a clean pre warmed slide (37ºC) and cover slip. The motility was determined by eye-estimation of the proportion of spermatozoa moving progressively straight forward at higher magnification (400X).

### Percentage of abnormality

For morphological assessment, a drop of diluted semen was placed on a slide and covered. A total of 200 sperm cells were counted on each slide. The morphology of the spermatozoa was assessed under phase contrast microscopy (magnification 1000X, oil immersion).

### Sperm Motility Recovery Rate

The sperm motility recovery rate was calculated by comparing the motility of pre freeze (Mpr) and post thaw (Mps) spermatozoa using the formula: Recovery rate = Mps/Mpr × 100% (Li *et al.*, 2005).

### Sperm progressive Motility Recovery Rate

The sperm progressive motility recovery rate was calculated by comparing the progressive motility of pre freeze (PMpr) and post thaw (PMps) spermatozoa. Recovery rate = PMps/PMpr × 100%.

### Sperm viability Recovery Rate

The sperm viability recovery rate was calculated by comparing the viability of pre freeze (Vpr) and post thaw (Vps) spermatozoa. Recovery rate = Vpr/Vps × 100%.

### Statistical analysis

The results were expressed as mean ± standard error of mean (S.E.M.). Means were analyzed by one-way analysis of variance, followed by the Tukey’s post hoc test to determine significant differences in all the parameters among all groups using the Mini tab statistical package.

Differences with values of *P*<0.05 were considered to be statistically significant.

## Results

The influence of vitamin B_12_ on the parameters of cooled semen, collected from *Dallagh rams*, was shown in Table 2.

**Table 2:** Motility, progressive motility, viability, abnormality and normal spermatozoa of *Dallagh* *rams*, stored at 5°C in diluents supplemented with different levels of vitamin B_12_ (mean ± S.E.M)

Groups	Motility (%)	Progressive motility (%)	Viability (%)	Normal spermatozoa (%)
Control	70.0^b^	64.8^b^	75.0^b^	91.0^b^
B_12_ (1 mg/ml)	75.0^ab^	69.7^ab^	80.0^ab^	94.0^ab^
B_12_ (2 mg/ml)	80.0^a^	75.0^a^	85.0^a^	95.8^a^
B_12_ (3 mg/ml)	77.0^a^	72.0^a^	82.0^a^	90.0^b^
SEM	± 1.60	± 1.45	± 1.50	± 1.129

Different superscript letters within the same column showed significant differences among the groups (*P*<0.05).

**Table 3:** Motility, progressive motility, viability, abnormal spermatozoa of *Dallagh* *rams* spermatozoa frozen in diluents supplemented with different levels of vitamin B_12_ (mean ± S.E.M)

Groups	Motility (%)	Progressive motility (%)	Viability (%)	Normal spermatozoa (%)
Control	13.0^c^	11.0^c^	18.0^c^	76.17^c^
B_12_ (1 mg/ml)	18.0^bc^	13.0^bc^	23.0^bc^	80.0^bc^
B_12_ (2 mg/ml)	32.0^a^	28.0^a^	38.0^a^	90.0^a^
B_12_ (3 mg/ml)	23.0^b^	18.0^b^	28.0^b^	85.0^ab^
SEM	±1.50	± 1.50	± 1.56	± 1.89

Different superscript letters within the same column showed significant differences among the groups (*P*<0.05).

**Table 4:** Recovery rate of motility, progressive motility and viability spermatozoa in *Dallagh* *rams*, in the presence and in the absence of vitamin B_12_.

Group	Recovery of Motility (%)	Recovery of Progressive motility (%)	Recovery of Viability (%)
Control	18.57^c^	16.97^c^	24.0^c^
B_12_ (1 mg/ml)	24.0^bc^	18.65^bc^	28.75^bc^
B_12_ (2 mg/ml)	40.0^b^	37.33^b^	44.70^b^
B_12_ (3 mg/ml)	29.87^a^	25.0^a^	34.14^a^
SEM	±1.63	±1.60	±1.70

Different superscript letters within the same column showed significant differences among the groups (*P*<0.05).

The highest motility (40% ± 1.63), progressive motility (37.33% ± 1.60) and viability recovery rate (44.70% ± 1.70) were observed in Tris extender containing 2 mg/ml of vitamin B_12_.

## Discussion

In this study, we investigated the effect of vitamin B_12_ on the characteristics of spermatozoa in *Dallagh rams* during cooling and freezing/thawing conditions. The results indicated that the addition of 2 mg/ml of vitamin B_12_ to Tris extender increased motility, progressive motility, viability and the number of normal spermatozoa. Our observations indicate that vitamin B_12_ was able to protect sperm during storage at 5°C and freezing conditions.

Similar results were obtained by Asadpour *et al*. (2012), who showed that addition of 2 mg/mL vitamin B_12_ into extender increased the sperm motility, viability, normal spermatozoa and the hypo osmotic swelling test (HOST) in Ghezel × Baluchi and Ghezel × Arkharmerino rams during storage at 5°C. Hu *et al*. (2009) also stated that semen frozen in extender supplemented with 2.50 mg⁄ml of vitamin B_12_ had a significant increase in sperm quality during post thawing.

Ha and Zhao (2003) reported that extender supplemented with vitamin B improved the quality of frozen-thawed semen during cryopreservation. Increased sensitivity to reactive oxygen species (ROS) is one of the major changes in the preservation of spermatozoa (Sikka, 1996).

Oxidative stress results in reduce levels of intracellular ATP which decreases sperm motility and also initiates lipid peroxidation in the polyunsaturated fatty acid rich sperm plasma membrane (Verma and Kanwar, 1998).

Such events have been associated with increased permeability, enzyme inactivation and production of spermicidal end products. Lipid peroxidation leads to abnormal acrosome reaction and loss of membrane fluidity and fertilizing potential of spermatozoa (Verma and Kanwar, 1998).

Cyanocobalamin (vitamin B_12_) is active during cellular replication and DNA synthesis and is already being used as a treatment for male infertility in humans (Eskenazi *et al.*, 2005). Additionally, Boxmeer *et al*. (2007) showed that there is a positive correlation between the total vitamin B_12_ concentration in seminal plasma and the spermatozoa concentration in semen. Supplementation of vitamin B_12_ decreases the amount of ROS produced from oxidative stress in human semen (Chen *et al.*, 2001a, b).

In our study, Vitamin B_12_ probably provided protection of sperm cells from morphological defects by preventing free radical oxygen from damaging sperms and exerted a protective effect, thereby preserving the metabolic activity and cellular viability of ram spermatozoa. Therefore, the deleterious effects of cryopreservation were decreased and semen quality improved pre and post freezing. Cai *et al*. (2004) showed that vitamin B_12_ could improve the motility of bovine spermatozoa during the freezing-thawing process, which is consistent with coenzyme A activity of vitamin B_12_.

Especially, reduction in glutathione has very important implications for the biological activities and metabolism of sperm cells and probably the increased motility, progressive motility and viability of spermatozoa due to the increased seminal plasma glutathione peroxidase, which would give protection against spontaneous lipid peroxidation (Hu *et al.*, 2009).

Ha and Zhao (2003) showed that glutamic oxaloacetic transaminase (GOT) emission of ram seminal plasma was significantly decreased when vitamin B_12_ was added to semen extender, and this might be an important factor in improving sperm motility during cryo-preservation. Neild *et al*. (2003) had indicated that GOT was released from seminal plasma during the freezing-thawing process and it damages the acrosome of sperm. The optimal vitamin B_12_ supplementation to the extender could prevent active forms of oxygen generation and membrane lipid peroxidation and scavenge against ROS. Therefore, the deleterious effects of cryopreservation were decreased and the percentages of membrane and acrosome integrity were improved after thawing of sperm (Hu *et al.*, 2011).

Yufan (1998) reported that the addition of vitamin B_12_ to diluted bull semen resulted in sperm activity, were improved and sperm deformity rate and the GOT activity both decreased significantly, prolonged the life time period of the sperm. It showed that vitamin B_12_ could protect the sperm form structural damages during semen freezing. Our results showed that the increasing the level of vitamin B_12_ could not improve some of the characteristics of spermatozoa during pre and post freezing. Hu *et al*. (2011) reported that higher concentration of vitamin B_12_ (3.75 mg/mL) exerts toxic effects on bull spermatozoa. However the exact mechanism of the negative effects of the higher doses of vitamin B_12_ on spermatozoa characteristics requires a detailed to be investigation.

## Conclusion

Based on the results of the present study, we conclude that supplementation of vitamin B_12_ in the semen extender significantly improved spermatozoa quality, viability, motility, progressive motility, normal spermatozoa and decrease of morphological defects obtained compared to the extender lacking vitamin B_12_. We determined that the optimum concentration of vitamin B_12_ in Tris extender for the semen of *Dallagh rams* is 2 mg/mL.

Further research and assays regarding the mitochondrial membrane potential of sperm, acrosomal integrity and lipid peroxidation of the membrane is needed to evaluate and understand that precise physiological role of vitamin B_12_ in reproduction.
